# *Ab initio* gene prediction for protein-coding regions

**DOI:** 10.1093/bioadv/vbad105

**Published:** 2023-08-10

**Authors:** Lonnie Baker, Charles David, Donald J Jacobs

**Affiliations:** Department of Bioinformatics and Genomics, University of North Carolina at Charlotte, NC 28223, United States; Department of Bioinformatics, The New Zealand Institute for Plant and Food Research, Lincoln 7608, New Zealand; Department of Physics and Optical Science, University of North Carolina at Charlotte, NC 28223, United States; UNC Charlotte School of Data Science, University of North Carolina at Charlotte, NC 28223, United States

## Abstract

**Motivation:**

*Ab initio* gene prediction in nonmodel organisms is a difficult task. While many *ab initio* methods have been developed, their average accuracy over long segments of a genome, and especially when assessed over a wide range of species, generally yields results with sensitivity and specificity levels in the low 60% range. A common weakness of most methods is the tendency to learn patterns that are species-specific to varying degrees. The need exists for methods to extract genetic features that can distinguish coding and noncoding regions that are not sensitive to specific organism characteristics.

**Results:**

A new method based on a neural network (NN) that uses a collection of sensors to create input features is presented. It is shown that accurate predictions are achieved even when trained on organisms that are significantly different phylogenetically than test organisms. A consensus prediction algorithm for a CoDing Sequence (CDS) is subsequently applied to the first nucleotide level of NN predictions that boosts accuracy through a data-driven procedure that optimizes a CDS/non-CDS threshold. An aggregate accuracy benchmark at the nucleotide level shows that this new approach performs better than existing *ab initio* methods, while requiring significantly less training data.

**Availability and implementation:**

https://github.com/BioMolecularPhysicsGroup-UNCC/MachineLearning.

## 1 Introduction

Automated annotation of DNA sequences is needed to keep pace with the massive level of sequence data being collected. There are many methods that use machine learning (ML) and informatic algorithms to identify features such as genes in unlabeled DNA sequences. ML-based methods that use hidden Markov models (HMMs) currently dominate the field, with well-known examples being Augustus (Stanke and Waack 2003) and Genscan (Burge and Karlin 1997) among many others (Guigó *et al.* 1992, Salzberg *et al.* 1999, Korf 2004). Methods such as GRAIL (Xu *et al.* 1996) and Orphelia (Hoff *et al.* 2008) employ a neural network (NN). Informatic algorithms, such as the discrete Fourier transform (DFT) (Vaidyanathan and Yoon 2002, Yin and Yau 2008, Ahmad *et al.* 2017) and autocorrelation functions (Fickett 1982) have shown the ability to locate coding regions to varying degrees. Automated gene prediction is one of the oldest branches of bioinformatics, and a complete list of methods goes beyond the scope of this article.

A survey of current methods shows that good accuracy is achieved when dealing with curated datasets from well-studied model organisms. For example, in the 2006 EGASP study, sensitivities (Sn) and specificities (Sp) at the nucleotide level of 94.56% and 92.78% were demonstrated by several methods (Guigó *et al.* 2006). However, the complexity of the problem has left a significant gap in accuracy when these methods are applied to nonmodel species, as shown in a 2020 study (Scalzitti *et al.* 2020). Methods that yielded Sn and Sp values above 90% in EGASP were unable to obtain average Sn or Sp values above 60%.

Homology-based methods that use protein, RNA-seq, or expressed sequence tag databases often yield good accuracy. These methods include GenomeScan (Yeh *et al.* 2001), GeneWise (Birney *et al.* 2004), and Augustus (Stanke *et al.* 2006). Unfortunately, homology-based methods do not find genes that are not phylogenetically close to known genes. This makes homology-based methods more prone to learning species-specific patterns. A recent study using the ORForise framework showed that homology-based gene prediction methods gave unpredictable results, even if the test genome and model genome were from similar organisms (Dimonaco *et al.* 2022). In contrast, *ab initio* methods rely on statistical properties extracted from the sequence to generate predictions (Guigó *et al.* 1992, Burge and Karlin 1997, Salzberg *et al.* 1999, Stanke and Waack 2003, Korf 2004). These methods typically outperform homology-based methods for nonmodel organisms (Dimonaco *et al.* 2022).

Greater accuracy occurs when species-specific patterns are learned, which requires well-annotated organisms to train on. The G3PO dataset was designed to test the accuracy of gene prediction methods against DNA from a wide range of nonmodel organisms (Scalzitti *et al.* 2020). However, this dataset does not include significant amounts of intergenic DNA. Nevertheless, the average accuracy of existing methods on the G3PO dataset is much lower than those reported on model organisms.

Typically, datasets used to test gene prediction methods remove the vast majority of intergenic DNA, restricting to small DNA regions known to contain one or more genes. For example, the dataset used in the EGASP study (Guigó *et al.* 2006) covered 1% of the human genome, with more than half of the sequences being 1 Mb long and containing experimentally verified genes. High accuracy has been achieved by several methods in this dataset, but the removal of large amounts of intergenic DNA reduces the rate of false positives and increases accuracy. If the test sequence is a whole chromosome or genome, the accuracy is invariably reduced.

No current method yields good specificity/sensitivity results over entire chromosomes, where the organisms used for training are not related to the test organisms. To address this challenge, we present a new *ab initio* ML method that uses a NN to learn patterns of features in labeled DNA to predict DNA coding locations in unlabeled sequences. The features are generated by a set of sensor algorithms that use a contiguous window of nucleotides as input and output a numerical value. Most sensors are based on previously developed informatic algorithms. This approach assigns to each nucleotide a vector of features that serve as input to a NN classifying each nucleotide as coding or noncoding, which improves on the sensor and NN-based method of Uberbacher and Mural (1991) and later implemented in the GRAIL algorithm (Xu *et al.* 1996). In addition, a novel postprocessing step is applied that improves accuracy by adjusting initial predictions using known genomic patterns: start codons, stop codons, and splice site motifs.

## 2 Methods

### 2.1 Feature generation using sensors

To quantify sequence characteristics, features are generated from the DNA sequence using 16 sensors, each describing a particular aspect of the local environment of a nucleotide (nt). Each sensor produces a single real numerical value. No reference is made to a reading frame. All sensors are confined to a window size of W=69 nt, where the query nt is at the center. Other window sizes were explored. For W≤99, the results were statistically equivalent, and for W>99, accuracy noticeably decreased as *W* increased. The algorithm for each sensor type is defined next.

**(1) Periodicity algorithm.** A DFT of DNA-coding regions is known to possess an enhanced period-3 signal when compared to noncoding regions (Vaidyanathan and Yoon 2002, Yin and Yau 2008, Ahmad *et al.* 2017). This signal is a result of the three letter codon set that translates to specific amino acids. To capture this periodicity, the first step requires translating the four bases {A, T, G, C} into a mathematical representation using an encoding scheme (Hota and Srivastava 2010, Das *et al.* 2017, Mabrouk 2017). Binary encoding, also known as Voss mapping, is used here for the periodicity sensor. The DNA sequence is transformed into a stack of four distinct binary sequences highlighting the location of a specific base through the following mapping: $A\to {\left[1000\right]}^{T}$, $T\to {\left[0100\right]}^{T}$, $G\to {\left[0010\right]}^{T}$, $C\to {\left[0001\right]}^{T}$. Indeterminate nucleotides such as N are assigned a null [0000] vector. Given the stacked binary string $x^{\left(j\right)}$ of four rows, the output for the *j*th stacked row is given as follows: where $x_{n}^{\left(j\right)}$ is the binary value of the element of the string *x* at position *n* for stack *j*. The magnitude of the resulting DFT signal at a frequency of $k=\frac{W}{3}$ is the period-3 signal. The DFT sensor output is given as follows:


(1) $$S_{k}^{\mathrm{DFT}}\left[x^{\left(j\right)}\right]=\left|\sum_{n=0}^{W-1}x_{n}^{\left(j\right)}\;e^{-i2\pi n\frac{k}{W}}\right|$$

(2) $$S^{\mathrm{DFT}}=\sum_{j=1}^{4}\left|\sum_{n=0}^{W-1}x_{n}^{\left(j\right)}\;e^{-i\frac{2}{3}\pi n}\right|.$$

**(2–6) k-mer frequency algorithms.** The frequencies of occurrence of specific nt words or k-mers in coding regions are known to be different than those in noncoding regions (Claverie *et al.* 1990, Damaševicius 2008). Sliding one base at a time within a window, each k-mer is associated with two frequency weight factors, where their ratio is used to differentiate different types of regions. At any location along the sequence, the frequency weight factors of k-mer *k* inside the coding and noncoding regions are, respectively, given by $p_{1}(j|k)$ and by $p_{0}(j|k)$. Here *j* runs from 1 to $4^{k}$ representing a distinct k-mer. The ratio of these frequency weight factors for the *j*th k-mer within a window is given by


(3) $$S_{k}(j)=-\ln\left(\frac{p_{1}(j|k)}{p_{0}(j|k)}\right)$$

For a window of size *W* nt and a k-mer size of *k*, a feature is calculated as the sum of all $S_{k}(j_{n})$ where $j_{n}$ specifies the specific k-mer found along the sequence at position *n*: where five k-mer sensors are implemented, requiring k={2,3,4,5,6} to be calculated.


(4) $$S_{k}^{K}=\sum_{n=1}^{W-k-1}S_{k}(j_{n})$$

Statistical weights are estimated from DNA sequences in the training sets. To achieve 1000 to 1 statistics, a number of sequences containing a total of $4^{k}\times k\times 1000$ bases is extracted from both coding and noncoding regions of the training set. The probabilities $p_{1}(j|k)$ and $p_{0}(j|k)$ are calculated by the total number of the *k*th k-mer divided by the total number of bases within the extracted sequences. When calculating $S_{k}^{K}$ of test sequences, the values of $p_{1}(j|k)$ and $p_{0}(j|k)$ from the training sequence are used, which are provided in Supplementary data as cvs files. Each cvs file contains the frequencies of all possible k-mers of a given size in both coding and noncoding regions. Maximum effectiveness of the k-mer sensors occur when each k-mer distribution for the training species is identical to that for the test species.

**(7) GC content algorithm.** Higher GC content has been shown to have a greater propensity for coding regions (Oliver and Marín 1996, Amit *et al.* 2012, Al-Ajlan and El Allali 2018). The GC content algorithm calculates the GC content of a sequence, denoted as $S^{gc}$. Let the numbers of each base inside a window be given by $n_{A}$, $n_{T}$, $n_{G}$, and $n_{C}$, then GC content is given by


(5) $$S^{gc}=\frac{n_{G}+n_{C}}{n_{A}+n_{T}+n_{G}+n_{C}}\;.$$

**(8–11) Start and stop codon distance algorithms.** Within a segment of DNA containing a gene, start and stop codons are located at natural boundaries between coding and noncoding regions. Moving in the forward direction from 5′ to 3′, a start codon marks a division point with noncoding sequence to the immediate left and coding sequence to the immediate right. Similarly, a stop codon marks a division point with coding sequence to the left and noncoding sequence to the immediate right. It stands to reason that there could be a statistically significant difference in the sequence composition of DNA which lies to the left or right of these codon motifs.

Four features are defined that take into consideration the nt distance on either side from the query nt to the nearest start and stop codon motifs. For a window size of *W* nt and distance *D* nt from the center of the window to the nearest start or stop codon motif, the feature output is given by


(6) $$S^{\mathrm{dis}}=\max\left(\frac{W}{2}-D,0\right)\;.$$

Although the identified motifs within a window are used to determine the distance *D*, they are generally not actual start and stop codons. Four start and stop codon features are generated for each sample base to (i) indicate the distance to the right of the nearest start codon; (ii) indicate the distance to the left of the nearest start codon; (iii) indicate the distance to the right of the nearest stop codon, and (iv) indicate the distance to the left of the nearest stop codon. Furthermore, when no start/stop codon motif is found, $S^{\mathrm{dis}}=0$.

**(12–15) Splice site distance algorithms.** The same method as the start/stop codon distance algorithm is applied to the nearest canonical splice sites. More than 98% of CDS subregions produced by splicing are bounded by canonical “YAG” acceptor sites on their left edges and canonical “GT” donor sites on their right edges (Frey and Pucker 2020). Four algorithms are implemented to track the left and right sides of the “YAG” acceptor site and the “GT” donor site.

**(16) Shannon entropy algorithm.** The 69 nt window is viewed as an emitter for a random sequence of four bases. Neglecting correlations, the entropy per base is calculated as follows: where P(b) is the probability of base *b* with b=A,T,G,C. The values of P(b) are calculated by counting the occurrence of each base inside the window. For example, $P(A)=n_{A}/W$. It has been shown that coding regions have less entropy than non-coding regions (Simões *et al.* 2021). Unlike k-mer sensors, the statistical weights for P(b) are determined based on the numbers of nucleotides observed within the window.


(7) $$S^{\mathrm{ent}}=\frac{-1}{W}\;\sum_{b}n_{b}\;P(b)\;\text{log}\;P(b)$$

### 2.2 Data preparation

Files containing DNA sequences in FASTA format and general feature format (GFF) files indicating known positions of CDS regions are used to construct training, validation and test sets. A nt located within a CDS region according to the GFF file is labeled as coding (1), otherwise as noncoding (0). Note that an nt belonging to CDS region that is optionally transcribed due to alternative splicing are given a coding label. To minimize labeling errors in supervised learning, four model species with well-annotated genomes were used for training and validation. Specifically, FASTA and GFF files for *Drosophila melanogaster* (assembly BDGP6.32), *Homo sapiens* (assembly GRCh38), *Mus musculus* (assembly GRCm39), and *Caenorhabditis elegans* (assembly WBcel235) were downloaded from Ensembl (available at https://ftp.ensembl.org/pub/release-103/fasta/ and http://ftp.ensembl.org/pub/release-103/gff3/).

Four training sets were generated from the following model species chromosomes: *D.melanogaster* chromosome 2 l, *H.sapiens* chromosome 21, *M.musculus* chromosome 19 and *C.elegans* chromosome I. Per chromosome, a test set is formed by sequestering the first and last 34 nt to ensure proper feature extraction from a 69 nt sensor window. Feature extraction algorithms are executed with the sensor window centered at each nt. The collected outputs of these algorithms form a 16 component vector to represent each nt in the test set.

As explained in the following, we train an NN on this training data one species at a time. As such, when we apply the trained NN model on a species different from the one it is trained on, there is no overlap between the test and training set. To maintain no overlap between training and testing sets when the training and test species are the same, the chromosomes used as test sets are as follows: *D.melanogaster* 2R, *H.sapiens* 20, *M.musculus* 18, and *C.elegans* II.

For a fifth test set, we use the G3PO dataset (Scalzitti *et al.* 2020) consisting of 1793 genes from 147 nonmodel organisms. Each sequence in the G3PO dataset represents a single gene that includes all exons and introns, augmented by short segments of intergenic DNA. This dataset was constructed to compare the top five *ab initio* gene prediction methods: Genscan (Burge and Karlin 1997), GlimmerHMM (Salzberg *et al.* 1999), GeneID (Guigó *et al.* 1992), Snap (Korf 2004), and Augustus (Stanke and Waack 2003), and is also used here to compare our sensor-NN approach to these methods.

### 2.3 Neural networks

A feed-forward, fully connected NN consisting of an input layer with 16 neurons (one for each sensor) followed by *h* hidden layers (each layer with $n_{h}$ neurons) and a two neuron output layer is implemented in Keras (Chollet *et al.* 2015). A large number of NN configurations were explored with *h* ranging from 1 to 10, and $n_{h}$ ranging from one to several hundred. Increasing the capacity of the NN did not increase accuracy beyond a single hidden layer of 10 neurons. This indicates that the combination of sensor algorithms provides features with differentiable properties for classification and additional latent features extracted with a deep NN does not increase accuracy. All neurons use SELU (scaled exponential linear unit) activation functions (Klambauer *et al.* 2017). The output of the final two neurons is not normalized and loss at each training epoch is calculated by sparse categorical cross-entropy from logits. Each of the two output neurons therefore represents one of the two possible classes (CDS or non-CDS). The predicted classification of each sample nt is determined by whichever output neuron has the highest output value.

Training sets are generated by selecting $N_{s}$-labeled nucleotides from a single chromosome. First, all CDS-labeled (assigned 1) nucleotides are grouped and $N_{1}$ of these are randomly selected without replacement. Second, all non-CDS-labeled (assigned 0) nucleotides are grouped, and $N_{0}$ of these are randomly selected without replacement. No distinction between intergenic or intron regions are made for non-CDS labels. Note that $N_{s}=N_{1}+N_{0}$. Training on imbalanced sets of coding versus noncoding samples was observed to increase sensitivity at the expense of specificity (or vice versa), but these trade-offs were deemed a net loss. Therefore, in this work $N_{1}=N_{0}=N_{s}/2$. We apply an 80/20 split between training and validation sets following common practice in training an NN. With the 80/20 split, each model (tied to a specific species) is trained using a 5-fold cross-validation scheme.

Output of the NN consists of predicted nucleotide labels. Each nt has either a positive (1) or negative (0) classification label. These predictions are referred to as *initial predictions* as they are the first set of predictions generated by this method. The CDS prediction algorithm which follows generates a set of *adjusted predictions* which are the actual output of the method.

### 2.4 CDS prediction

In eukaryotic organisms, with few exceptions, the CDS regions that make up genes are bounded by highly conserved motifs. A gene composed of a single CDS begins with an ATG start codon followed by an integer number of three letter codons, and ends with one of the three possible stop codons, {TAG, TAA, TGA}. All prokaryotic genes belong to this single CDS form. The genes of eukaryotic organisms are usually composed of several CDS regions, which are combined by splicing before translation. A multiple CDS gene consists of three types of CDS regions, referred to as “start,” “internal,” and “stop” CDS regions. A “start” CDS begins with an ATG start codon, followed by a number of bases and usually terminating in a canonical GT donor splice site. A typical “internal” CDS is bounded by the canonical YAG acceptor and GT donor splice sites. An “end” CDS begins with an YAG acceptor site and terminates with a stop codon.

Once *initial predictions* from the NN have been generated for a sequence indicating a coding or noncoding label for each nucleotide, a CDS prediction algorithm is used to revise the predicted CDS regions to ensure proper boundary motifs. Since the NN predictions are not constrained to be self-consistent within a potential CDS region, these errors will be corrected. A *potential CDS* is defined as any region of DNA which is bounded by the proper motif sites described above.

**Figure 1.**
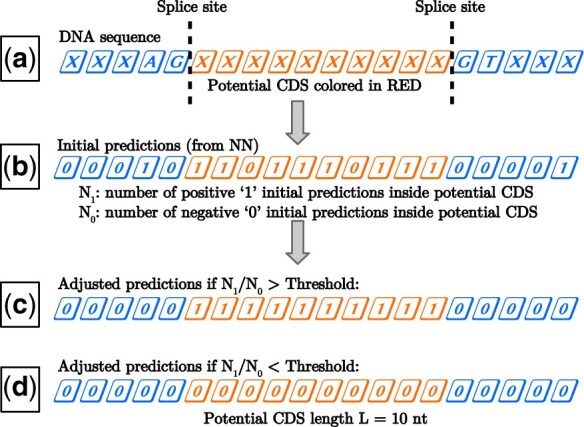
Overview of the CDS prediction algorithm. (a) A sample DNA sequence is shown to illustrate the location of splice sites. The sequence between these sites is a potential CDS. (b) The initial predictions generated by the NN consist of nucleotide level classifications. Within a potential CDS, the number of initial coding predictions (denoted by 1) is $N_{1}$ while the number of noncoding predictions (denoted by 0) is $N_{0}$. (c) If the $N_{1}/N_{0}$ ratio is above a user defined CDS/non-CDS threshold, all nts inside the potential CDS are converted to coding predictions by flipping all 0 to 1. (d) If the $N_{1}/N_{0}$ ratio is below the threshold, all nts inside the potential CDS are converted to noncoding predictions by flipping all 1 to 0. This cooperative transformation yields biologically possible predictions.

Using the locations of CDS boundary motifs, a list of all potential CDS regions is generated. While extremely short and long CDS regions are found in nature, a minimum and maximum potential CDS length can be set by the user. Limiting the minimum and maximum potential CDS lengths to reasonable numbers reduces computation time and has little impact on accuracy because extremely short and long CDS regions are rare (Long and Deutsch 1999). Figure 1 shows an example of a potential CDS of length L=10 nts. Only CDS regions for which $L_{\text{min}}<L<L_{\text{max}}$ are considered.

Within each potential CDS region, the initial predictions are processed as follows. Let $N_{1}$ equal the number of predicted coding nucleotides and $N_{0}$ equal the number of predicted noncoding nucleotides. The ratio $N_{1}/N_{0}$ is assigned to every potential CDS region. To generate a set of adjusted predictions, a user defined CDS/non-CDS threshold is chosen. All potential CDS regions for which $N_{1}/N_{0}$ is above this threshold are adjusted so that all nucleotides within the potential CDS are converted to coding predictions. Potential CDS regions for which the $N_{1}/N_{0}$ ratio is below the threshold are converted to noncoding predictions. Labels for regions which lie outside a potential CDS are also converted to noncoding. This process, which creates cooperativity in the form of consensus, is illustrated in Fig. 1.

Overlapping CDS regions may be generated by the above process when a short potential CDS region shares a start codon, stop codon, or splice site with a larger potential CDS region. In this situation, the largest overlapping region for which $N_{1}/N_{0}$ is above the threshold takes precedence. In other words, once a large potential CDS region has been found for which $N_{1}/N_{0}$ is above the threshold, any smaller potential CDS regions which are contained within the larger CDS region are no longer considered. Operationally, the tests start at the largest potential CDS region up to $L_{\text{max}}$ and the tests occur at each successively smaller potential CDS region until a success is found or until $L_{\text{min}}$ is reached. In the current study, potential CDS regions which were between 40 and 400 nts long were considered. These numbers were chosen so as to exclude very few exons based on studies of exon length distributions (Long and Deutsch 1999).

The value of the CDS/non-CDS threshold affects sensitivity and specificity. Decreasing the threshold increases sensitivity and reduces specificity by simultaneously increasing the number of true and false positives. As the CDS/non-CDS threshold increases, the number of true and false positives decreases. As shown in results, a net gain in sensitivity and specificity compared to the raw NN predictions is achieved through this postprocessing. Two heuristic methods are presented to set the CDS/non-CDS threshold for two distinct situations that arise in gene prediction: an assembled genome for an organism or meta-genomic data consisting of many unassembled sequences.

The percentage of protein coding DNA in the genomes of many organisms is known to be low. In humans, the coding fraction of 0.03 or less is observed on each chromosome (Ahnert *et al.* 2008). Most other mammals have a low coding faction. An estimate of the best CDS/non-CDS threshold can be made by observing the total fraction of test DNA which is predicted as coding. For example, if the fraction of coding DNA on human chromosome 21 was predicted to be 0.25, the method over-predicts CDS regions and generates many false positives. By increasing the CDS/non-CDS threshold, a higher level of evidence is required before predicting a CDS region. This results in fewer predicted CDS regions and a lower predicted fraction of coding DNA.

A sweep over CDS/non-CDS thresholds is carried out, starting low and gradually increasing. At each iteration, all potential CDS regions are evaluated as either true or false. The total fraction of the test DNA predicted as coding is then calculated by dividing the number of nucleotides inside true CDS regions, divided by the number of nucleotides in the test sequence. If this fraction is significantly above the expected level, the CDS/non-CDS threshold is increased again and the process repeats. Once a set of CDS predictions are made for which the fraction of the predicted coding nucleotides is close (typically above) to what is expected, the process is complete and the current predictions are accepted.

Test sequences from unassembled reads or samples from diverse species will generally lack a representative fraction of coding nucleotides of a parent species. In this case, an educated guess must be made when setting a CDS/non-CDS threshold. Accuracy results for a sweep of CDS/non-CDS threshold values have been collected over sequences from various species. Results indicate that any reasonable CDS/non-CDS threshold improves the raw NN results.

### 2.5 Accuracy measures

We calculate sensitivity as: Sn=TP/(TP+FN), specificity as Sp=TN/(TN+FP) and F1=2TP/(2TP+FP+FN), where TP, FN, TN, and FP are respectively the counts of true positives, false negatives, true negatives and false positives. To directly compare to literature results, we calculate precision, defined as PPV=TP/(TP+FP), the arithmetic mean of Sn and PPV defined as MSP=(Sn+PPV)/2 and the harmonic mean of Sn and PPV, which equals the F1 measure.

## 3 Results

### 3.1 Training results

Five NNs are trained on each of the four model species (20 NNs total). We considered different training set sizes. Our largest training set has $N_{s}=10^{7}$ nts where we train on 8M nts, with 2M nts used in a 5-fold cross-validation scheme. All results presented here are from models using the 10M nt training set. Results obtained when using a 1M nt training set are given in Supplementary data. Diminishing returns in accuracy improvement begin before reaching 1M training samples. However, using 10M nt for training yields a slightly better result, although not statistically significant based on a *P*-value test using a .05 threshold. Since a fixed NN model is expected to make useful predictions over entire chromosomes for an arbitrary number of species, it is desirable to achieve a useful model when the amount of training data is much less than the test data, which is the case considered here. The ability to train an accurate NN model using 1M nt is a strength of the sensor-NN approach, indicating generalizability.

### 3.2 Whole chromosome test results

Table 1 gives nucleotide level sensitivity (Sn), specificity (Sp), and balanced accuracy (BA) results from trained NNs before applying the CDS algorithm. The listed numbers in the table reflect the mean value across all five NN models. In all cases, the standard deviation across five models is 0.02 or less.

**Table 1.** Nucleotide level sensitivity (Sn), specificity (Sp), and balanced accuracy (BA) results from trained NNs before adjustments from CDS prediction algorithm.

	*D.mel.*	*H.sap.*	*M.mus.*	*C.ele.*
	(test)	(test)	(test)	(test)
*D.melanogaster*	Sn: 0.78	Sn: 0.83	Sn: 0.75	Sn: 0.47
(training)	Sp: 0.84	Sp: 0.53	Sp: 0.65	Sp: 0.9
	BA: 0.81	BA: 0.68	BA: 0.7	BA: 0.68
*H.sapiens*	Sn: 0.63	Sn: 0.94	Sn: 0.67	Sn: 0.73
(training)	Sp: 0.78	Sp: 0.61	Sp: 0.78	Sp: 0.65
	BA: 0.7	BA: 0.77	BA: 0.73	BA: 0.69
*M.musculus*	Sn: 0.85	Sn: 0.45	Sn: 0.75	Sn: 0.65
(training)	Sp: 0.64	Sp: 0.95	Sp: 0.82	Sp: 0.75
	BA: 0.74	BA: 0.7	BA: 0.78	BA: 0.7
*C.elegans*	Sn: 0.74	Sn: 0.84	Sn: 0.65	Sn: 0.84
(training)	Sp: 0.72	Sp: 0.46	Sp: 0.66	Sp: 0.75
	BA: 0.73	BA: 0.65	BA: 0.66	BA: 0.8

Training species are indicated by row and test species by column. Mean values across all 16 cases: Sn = 0.72, Sp = 0.72, BA = 0.72.

After obtaining initial nucleotide level predictions from the NN, CDS regions are predicted by increasing the CDS/non-CDS threshold until the predicted coding fraction is below an estimate of the known coding fraction of a similar species. The coding fraction is the fraction of nucleotides which belong to coding regions inside a sequence. For the results given in Table 2, the target coding fractions are as follows: *D.melanogaster*: 0.2, *H.sapiens*: 0.1, *M.musculus*: 0.1, and *C.elegans*: 0.4. The listed numbers in Table 2 reflect the mean value across all five NN models after the CDS algorithm is applied. In all cases, the standard deviation across five models is 0.02 or less. Supplementary Figs 1 and 2 show Sn and Sp as a function of CDS/non-CDS threshold, respectively, for the 1M and 10M datasets. The corresponding Tables 1 and 2 are shown as Supplementary Figs S3 and S4 respectively for the 1M training set.

**Table 2.** Nucleotide level sensitivity (Sn), specificity (Sp), and balanced accuracy (BA) results from trained NNs after the CDS algorithm processing.

	*D.mel.*	*H.sap.*	*M.mus.*	*C.ele.*
	(test)	(test)	(test)	(test)
*D.melanogaster*	Sn: 0.82	Sn: 0.78	Sn: 0.61	Sn: 0.78
(training)	Sp: 0.87	Sp: 0.71	Sp: 0.81	Sp: 0.73
	BA: 0.84	BA: 0.74	BA: 0.71	BA: 0.76
	Th: 10	Th: 18	Th: 29	Th: 1
*H.sapiens*	Sn: 0.66	Sn: 0.77	Sn: 0.58	Sn: 0.76
(training)	Sp: 0.83	Sp: 0.75	Sp: 0.88	Sp: 0.68
	BA: 0.74	BA: 0.76	BA: 0.73	BA: 0.72
	Th: 4	Th: 10	Th: 4	Th: 2
*M.musculus*	Sn: 0.88	Sn: 0.61	Sn: 0.93	Sn: 0.76
(training)	Sp: 0.56	Sp: 0.92	Sp: 0.5	Sp: 0.67
	BA: 0.72	BA: 0.76	BA: 0.72	BA: 0.72
	Th: 29	Th: 24	Th: 29	Th: 23
*C.elegans*	Sn: 0.63	Sn: 0.73	Sn: 0.46	Sn: 0.89
(training)	Sp: 0.84	Sp: 0.62	Sp: 0.81	Sp: 0.71
	BA: 0.74	BA: 0.68	BA: 0.64	BA: 0.8
	Th: 21	Th: 29	Th: 25	Th: 5

In addition, the CDS/non-CDS thresholds used to obtain the results shown is given. Training species are indicated by row and test species by column. Mean values across all 16 cases: Sn = 0.73, Sp = 0.74, BA = 0.74.

### 3.3 G3PO test results

We applied the same 20 distinct trained NNs from above to every gene in the G3PO dataset. An optimal CDS/non-CDS threshold should be independently set for each gene. However, in the analysis presented here, results are presented in three ways. First, a fixed threshold is set to identify a worse-case scenario. Next, we separately optimize on the MSP and F1 measures, which provide two different best case scenarios. The three ways of reporting results are reasonable to compare against the five state-of-the-art methods that were applied to the G3PO dataset.

**Figure 2.**
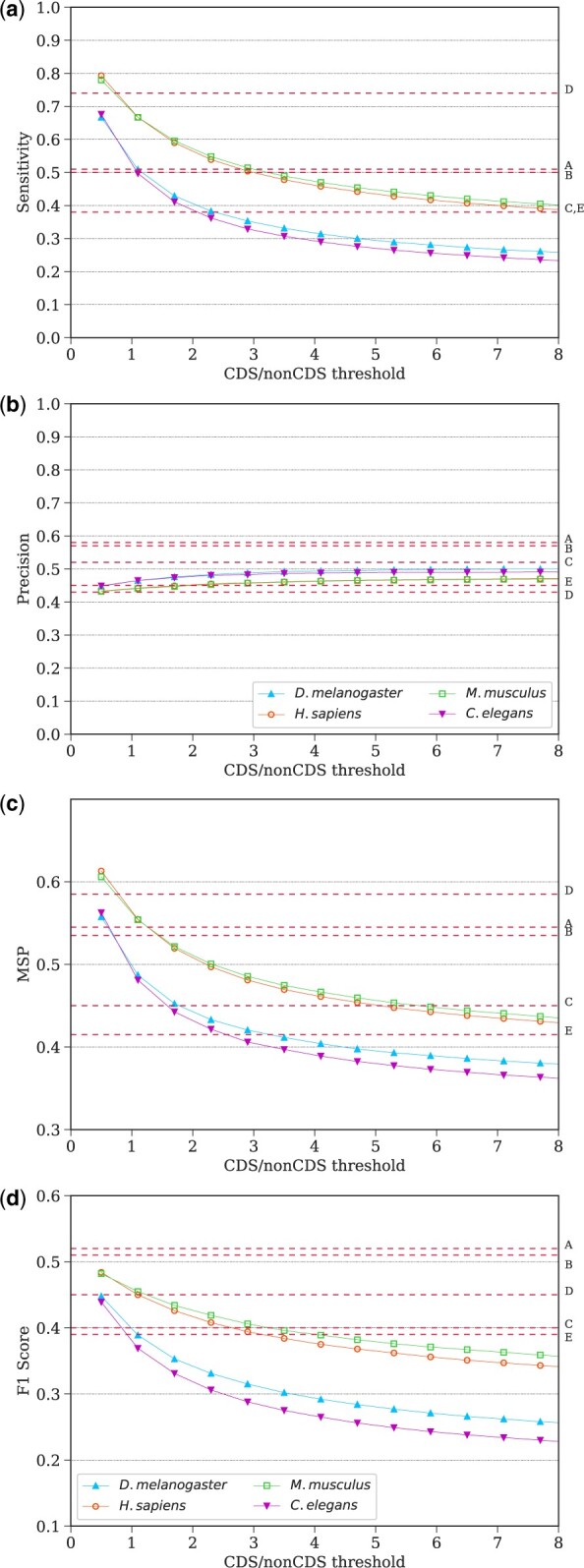
From top to bottom panels: (a) mean nucleotide level sensitivity (Sn), (b) precision (PPV), (c) arithmetic mean of Sn and PPV (MSP) and (d) the F1 score as functions of CDS/non-CDS threshold over the G3PO dataset. Training DNA species is indicated in the legend. The optimal values from the five top performing methods are shown by horizontal lines: AUGUSTUS (A), Genscan (B), GeneID (C), GlimmerHMM (D) and Snap (E).

In Fig. 2, Sn, PPV, MSP, and F1 are shown as a function of the CDS/non-CDS threshold ranging from 0.5 to 8, where the same threshold is applied across all genes. There is a narrow range of CDS/non-CDS thresholds for which both Sn and PPV values are competitive to those produced by the five *ab initio* methods tested in Scalzitti *et al.* (2020), shown in Fig. 2 as labeled horizontal lines. For example, using a fixed CDS/non-CDS threshold of 0.7, our method yields a Sn of 0.74±0.02 and PPV of 0.44±0.02 trained on *H.sapiens* DNA. Importantly, this result is achieved using a fixed threshold, which puts the sensor-NN method at a disadvantage.

Our sensor-NN method is optimized for a single-species chromosome, with a species-specific CDS/non-CDS threshold. Table 3 summarizes upper bound estimates obtained by using a variable threshold per gene to maximize either MSP or F1 per species, along with the fixed threshold case.

**Table 3.** Summary of Sn, PPV, MSP, and F1 on the GP3O dataset for the five top performing methods and the sensor-NN method for three cases: when the CDS/non-CDS threshold is fixed at 0.7 or set to maximize MSP or F1 scores.

	Sn	PPV	MSP	F1	Th	SD
AUGUSTUS	0.51	0.58	0.55	0.52	NA	NA
Genscan	0.50	0.57	0.54	0.51	NA	NA
GeneID	0.38	0.52	0.45	0.40	NA	NA
GlimmerHMM	0.74	0.43	0.56	0.45	NA	NA
Snap	0.38	0.45	0.42	0.39	NA	NA
*D.melanogaster* MSP	0.69	0.50	0.59	0.47	1.75	4.88
*D.melanogaster* F1	0.67	0.54	0.60	0.51	3.41	5.73
*D.melanogaster* fixed	0.60	0.45	0.53	0.43	0.7	0
*H.sapiens* MSP	0.84	0.48	0.66	0.53	2.11	5.34
*H.sapiens* F1	0.71	0.53	0.62	0.54	3.75	5.85
*H.sapiens* fixed	0.74	0.44	0.59	0.47	0.7	0
*M.musculus* MSP	0.80	0.48	0.64	0.52	2.36	5.84
*M.musculus* F1	0.83	0.42	0.63	0.55	5.31	6.71
*M.musculus* fixed	0.74	0.44	0.59	0.47	0.7	0
*C.elegans* MSP	0.71	0.50	0.61	0.47	1.48	4.47
*C.elegans* F1	0.66	0.54	0.60	0.49	2.93	5.36
*C.elegans* fixed	0.60	0.46	0.53	0.41	0.7	0

The mean CDS/non-CDS threshold and standard deviation are also reported for the sensor-NN method.

## 4 Discussion

Our results show that without using phylogenetically close species to train on, ML predictions for coding nucleotides are obtained with an overall accuracy that is better than current state-of-the-art accuracy from models that train on phylogenetically close species. For example, when presented with an unlabeled *H.sapiens* chromosome 21, a sensitivity of 0.83 and specificity of 0.53 was achieved when a sensor-NN model was trained on DNA from *D.melanogaster* chromosome 2 L as summarized in Table 1. We found that the NN need not be sophisticated, suggesting that the 16 sensors generate a combined set of features that produce strong indicators for classification. This is not to say that adding more sensors could make the approach stronger and more robust.

After the ML step is finished, the postprocessing step is applied that introduces cooperative consensus building with a CDS/non-CDS threshold. Our experimentation showed that the CDS/non-CDS threshold should be set at a level that produces a fraction of predicted coding DNA slightly above the empirical CDS region content estimate. As a rule of thumb, the predicted fraction of coding DNA should be about 0.1 higher than the empirical CDS region content. For example, again considering *H.sapiens* chromosome 21 when a sensor-NN model was trained on DNA from *D.melanogaster*. Now, after the postprocessing step, we obtain a sensitivity of 0.78 and specificity of 0.71 choosing an appropriate threshold selected based on target coding fraction of 10%.

In the before/after example considered for model species, BA changes from 0.68 to 0.74, which is a net increase. Often there is an increase in BA, but it can decrease or have a modest increase. Moreover, high/low values of Sp and Sn often swap positions. These differences can be seen by comparing Tables 1 and 2 carefully. Nevertheless, even when BA decreases, the benefit of using the postprocessing CDS algorithm is that the predictions are biologically consistent in that all predicted CDS regions are bounded by legitimate motifs such as start and stop codons or splice site motifs (YAG or GT).

When dealing with unassembled or partially assembled genetic data, the CDS/non-CDS threshold should be set to a value that was successful on a model organism similar to the test organism when possible. If analyzing DNA from an unknown mammal, a model trained on human DNA with a CDS/non-CDS threshold set to four as suggested in Table 2. If no model organism is similar to a test organism, a CDS/non-CDS threshold near 1 yields results with relatively good accuracy.

The G3PO dataset was developed to test the effectiveness of modern gene prediction methods against various nonmodel organisms (Scalzitti *et al.* 2020). These recently published findings showed that the average accuracy of gene prediction methods in nonmodel organisms is low. It is important to note that the authors in that work used sensitivity and *precision* (but labeled as specificity) and F1 scores as their measures, which we followed suit to make direct comparisons. We make no claim as to whether Sp or PPV is intrinsically better. When training Genscan, GlimmerHMM, GeneID, Snap, and Augustus, the authors of the G3PO dataset selected organism-specific models that were phylogenetically similar to each test gene. In contrast, the training species we have used are generally not phylogenetically similar.

With one model species for training, our method was more accurate than many other methods that train on a larger pool of data from multiple species. Moreover, for the G3PO dataset, a sweep over the CDS/non-CDS thresholds for the CDS prediction algorithm reveals 0.7 is a good threshold when no information is available. We expect greater accuracy will be achieved in the G3PO dataset by setting the target CDS/non-CDS threshold to a relevant value for each species. Nevertheless, this test shows that in situations where little is known about the genome being studied, the true CDS/non-CDS threshold is not critical to obtain a prediction accuracy better than current state-of-the-art methods.

For the 1M nt and the 10M nt training sets, we show Supplementary Figs 5 and 6 to respectively display the distribution of variable thresholds that were obtained by maximizing the MSP accuracy. Similarly, Supplementary Figs 7 and 8 show the distribution of variable thresholds obtained by maximizing the F1 score. Supplementary Figs 9 and 10 respectively show the distribution of a fixed threshold when Sp and Sn are made equal. In all cases, the distribution is sharply peaked at low values of the threshold. However, a heavy tail is also observed in all cases, indicating that larger thresholds are required.

## 5 Conclusion

Obtaining good accuracy from gene prediction methods on highly curated and annotated datasets of model organisms is a poor indication of their accuracy when applied to entire chromosomes from nonmodel organisms. We have implemented a NN approach with novel sensor-based features as input that predicts protein-coding nucleotides across entire chromosomes using a relatively small training set. Furthermore, we include a postprocessing step that enforces biological constraints to be satisfied through a novel cooperative consensus procedure. This method greatly improves accuracy under realistic conditions of a newly assembled genome from a nonmodel organism, where locations of genes are not known, and homology data from a phylogenetically close organism need not be available. The framework of this method lends itself to the development of new sensor algorithms and the evaluation of optimal feature sets to further increase accuracy.

Our results suggest that future work can focus on identifying additional sensors that can be included in this approach. It is worth mentioning that we initially thought that training on a specific species would limit the applicability of the NN to that species or closely related species. However, we have shown that the differences in the trained NN can be compensated by adjusting the CDS/non-CDS threshold, making the procedure a general and powerful approach. These findings also suggest that cross-species training of the NN could be an interesting area for exploration. Another possible way to enhance accuracy is to use an ensemble of NNs trained on different species. Overall, our results suggest that the sensor-NN idea, originally put forth by Uberbacher and Mural (1991), has much potential for growth.

## Supplementary Material

vbad105_Supplementary_Data
