# Supplementary material for: Ab initio gene prediction for protein-coding regions
Source: Bioinform Adv. 2023 Aug 10;3(1):vbad105. doi: 10.1093/bioadv/vbad105 (PMC10448985; doi:10.1093/bioadv/vbad105)
Supplement: vbad105_Supplementary_Data [file vbad105_supplementary_data.pdf]

Supplementary Information for

Ab initio gene prediction for protein coding regions

Lonnie Baker, Charles David and Donald J. Jacobs

Donald J. Jacobs.
E-mail: djacobs1@uncc.edu

This PDF file includes:

Supplementary Figures S1 - S10

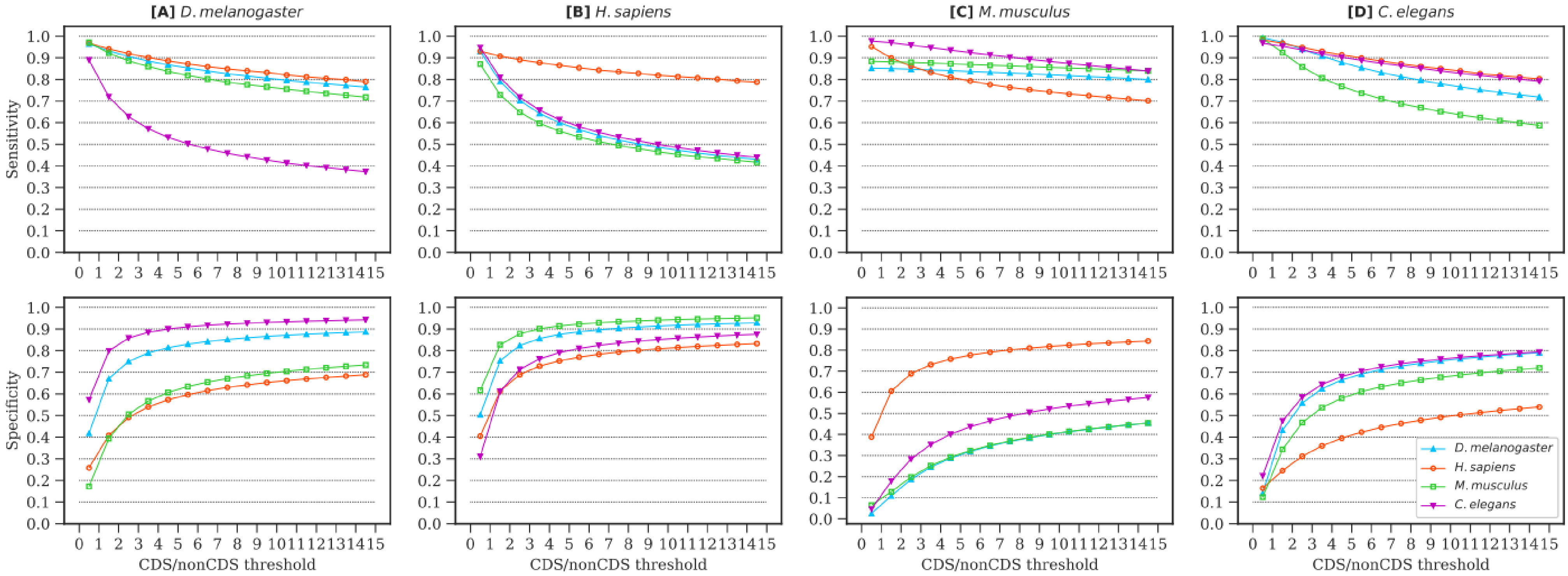

Fig. S1. Nucleotide level accuracy results over chromosomes: *D. melanogaster* (2L), *H. sapiens* (21), *M. musculus* (19) and *C. elegans* (I). Training species is indicated at the top of each pair of plots. Training consisted of 800,000 randomly selected nts with a 50/50 split of coding and non-coding samples.

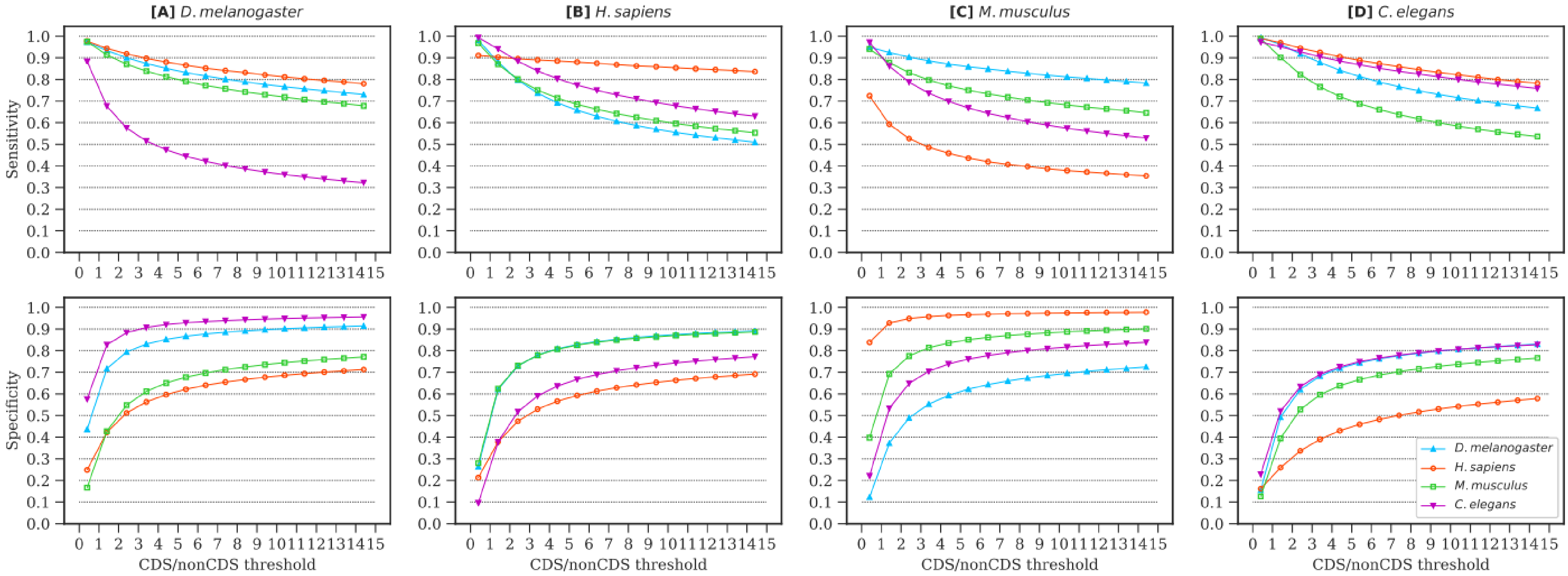

Fig. S2. Nucleotide level accuracy results over chromosomes: *D. melanogaster* (2L), *H. sapiens* (21), *M. musculus* (19) and *C. elegans* (I). Training species is indicated at the top of each pair of plots. Training consisted of 8 million randomly selected nts with a 50/50 split of coding and non-coding samples.

	D. mel. (test)	*H. sap.* (test)	*M. mus.* (test)	*C. ele.* (test)
D. melanogaster (training)	Sn:0.9<br>Sp:0.69<br>BA:0.8	Sn:0.85<br>Sp:0.51<br>BA:0.68	Sn:0.79<br>Sp:0.61<br>BA:0.7	Sn:0.52<br>Sp:0.88<br>BA:0.7
H. sapiens (training)	Sn:0.56<br>Sp:0.85<br>BA:0.7	Sn:0.93<br>Sp:0.56<br>BA:0.75	Sn:0.55<br>Sp:0.88<br>BA:0.72	Sn:0.58<br>Sp:0.78<br>BA:0.68
M. musculus (training)	Sn:0.96<br>Sp:0.39<br>BA:0.67	Sn:0.78<br>Sp:0.75<br>BA:0.77	Sn:0.93<br>Sp:0.51<br>BA:0.72	Sn:0.9<br>Sp:0.48<br>BA:0.69
C. elegans (training)	Sn:0.78<br>Sp:0.68<br>BA:0.73	Sn:0.86<br>Sp:0.49<br>BA:0.68	Sn:0.69<br>Sp:0.62<br>BA:0.66	Sn:0.8<br>Sp:0.77<br>BA:0.78

Fig. S3. Nucleotide level Sn, Sp and BA scores from neural networks trained on 800,000 samples but before the CDS prediction step is implemented. Mean values: Sn = 0.77, Sp = 0.65, BA = 0.71.

	D. mel. (test)	*H. sap.* (test)	*M. mus.* (test)	*C. ele.* (test)
D. melanogaster (training)	Sn:0.78<br>Sp:0.75<br>BA:0.76<br>Th: 13	Sn:0.78<br>Sp:0.7<br>BA:0.74<br>Th: 18	Sn:0.61<br>Sp:0.8<br>BA:0.7<br>Th: 29	Sn:0.81<br>Sp:0.71<br>BA:0.76<br>Th: 1
H. sapiens (training)	Sn:0.66<br>Sp:0.85<br>BA:0.76<br>Th: 4	Sn:0.82<br>Sp:0.67<br>BA:0.74<br>Th: 20	Sn:0.58<br>Sp:0.91<br>BA:0.74<br>Th: 4	Sn:0.76<br>Sp:0.67<br>BA:0.72<br>Th: 2
M. musculus (training)	Sn:0.76<br>Sp:0.53<br>BA:0.64<br>Th: 29	Sn:0.6<br>Sp:0.9<br>BA:0.75<br>Th: 24	Sn:0.75<br>Sp:0.62<br>BA:0.68<br>Th: 20	Sn:0.76<br>Sp:0.66<br>BA:0.71<br>Th: 23
C. elegans (training)	Sn:0.63<br>Sp:0.84<br>BA:0.74<br>Th: 21	Sn:0.73<br>Sp:0.6<br>BA:0.66<br>Th: 29	Sn:0.46<br>Sp:0.8<br>BA:0.63<br>Th: 25	Sn:0.85<br>Sp:0.72<br>BA:0.78<br>Th: 4

Fig. S4. Nucleotide level Sn, Sp and BA scores from neural networks trained on 800,000 samples after the CDS prediction step is implemented. Mean values: Sn = 0.71, Sp = 0.73, BA = 0.72.

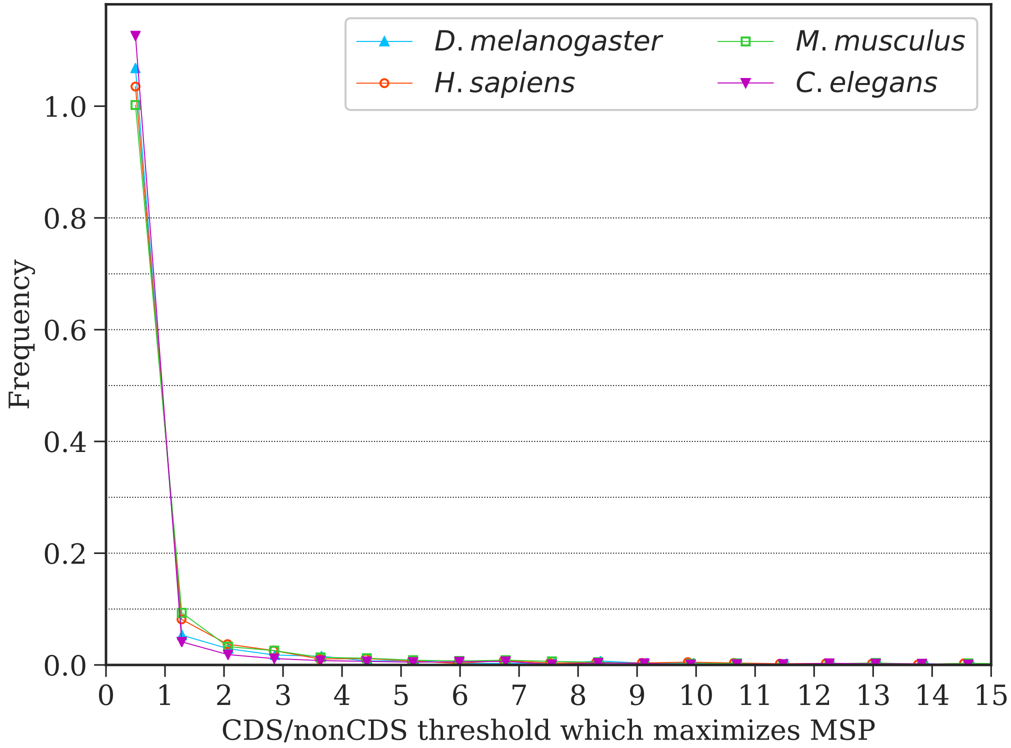

Fig. S5. Distribution of CDS/nonCDS threshold values which maximize the mean of sensitivity and precision (MSP) scores on individual genes from the G3PO dataset. Training species is indicated at the top of each pair of plots. Training consisted of 800,000 randomly selected nts with a 50/50 split of coding and non-coding samples.

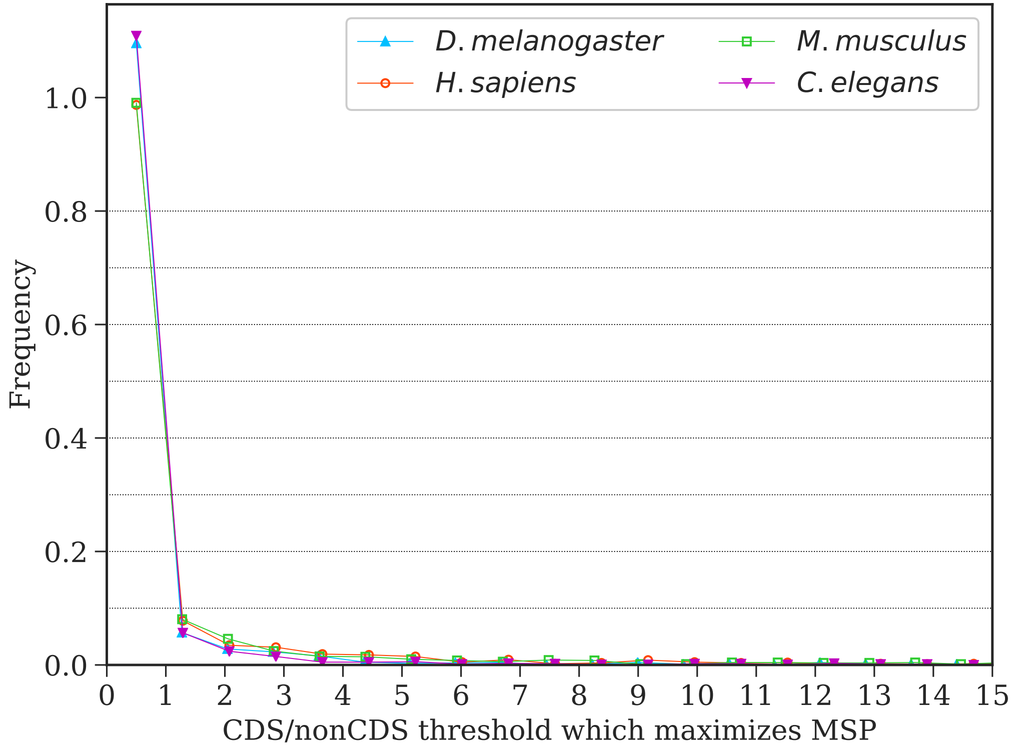

Fig. S6. Distribution of CDS/nonCDS threshold values which maximize the mean of sensitivity and precision (MSP) scores on individual genes from the G3PO dataset. Training species is indicated at the top of each pair of plots. Training consisted of 8 million randomly selected nts with a 50/50 split of coding and non-coding samples.

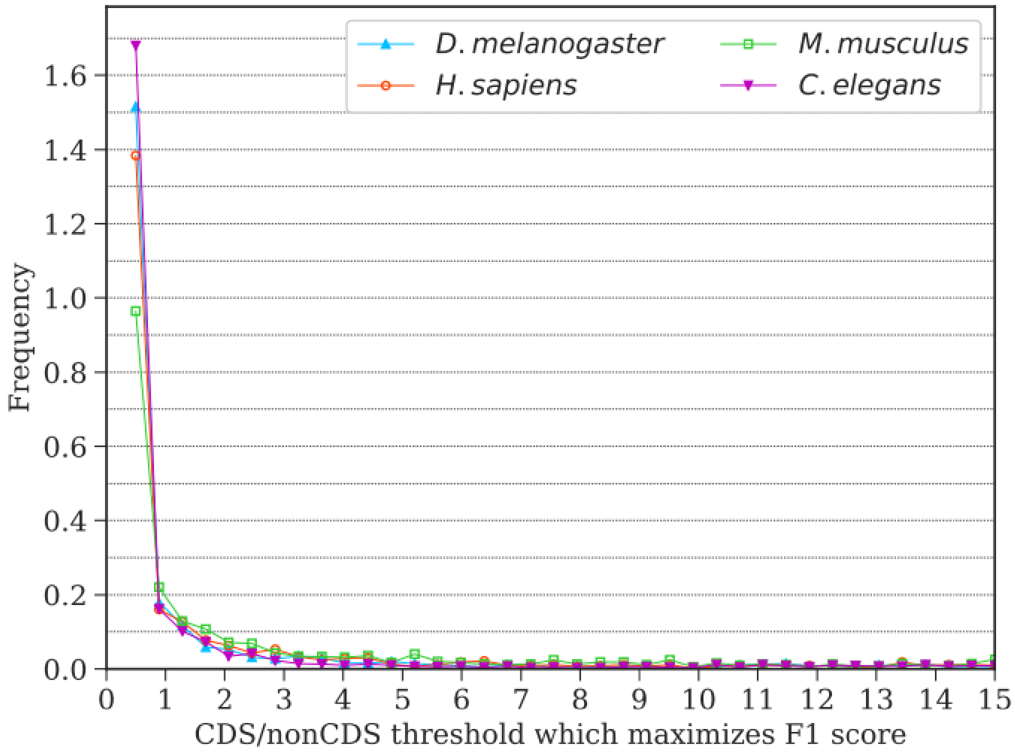

Fig. S7. Distribution of CDS/nonCDS threshold values which maximize F1 scores on individual genes from the G3PO dataset. Training species is indicated at the top of each pair of plots. Training consisted of 800,000 randomly selected nts with a 50/50 split of coding and non-coding samples.

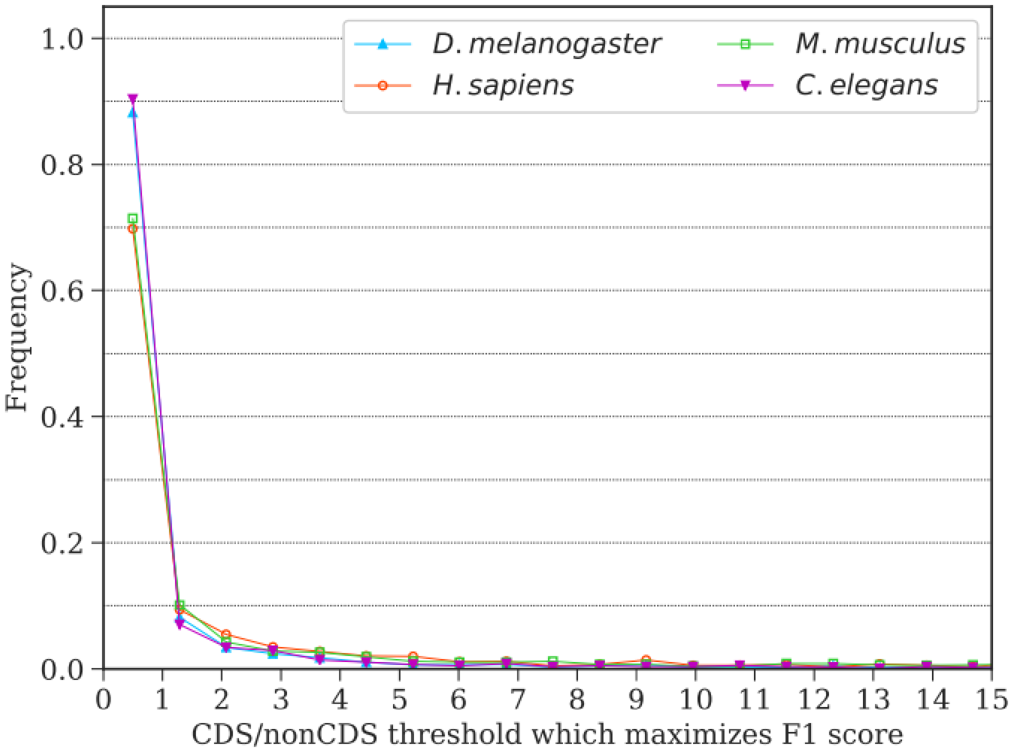

Fig. S8. Distribution of CDS/nonCDS threshold values which maximize F1 scores on individual genes from the G3PO dataset. Training species is indicated at the top of each pair of plots. Training consisted of 8 million randomly selected nts with a 50/50 split of coding and non-coding samples.

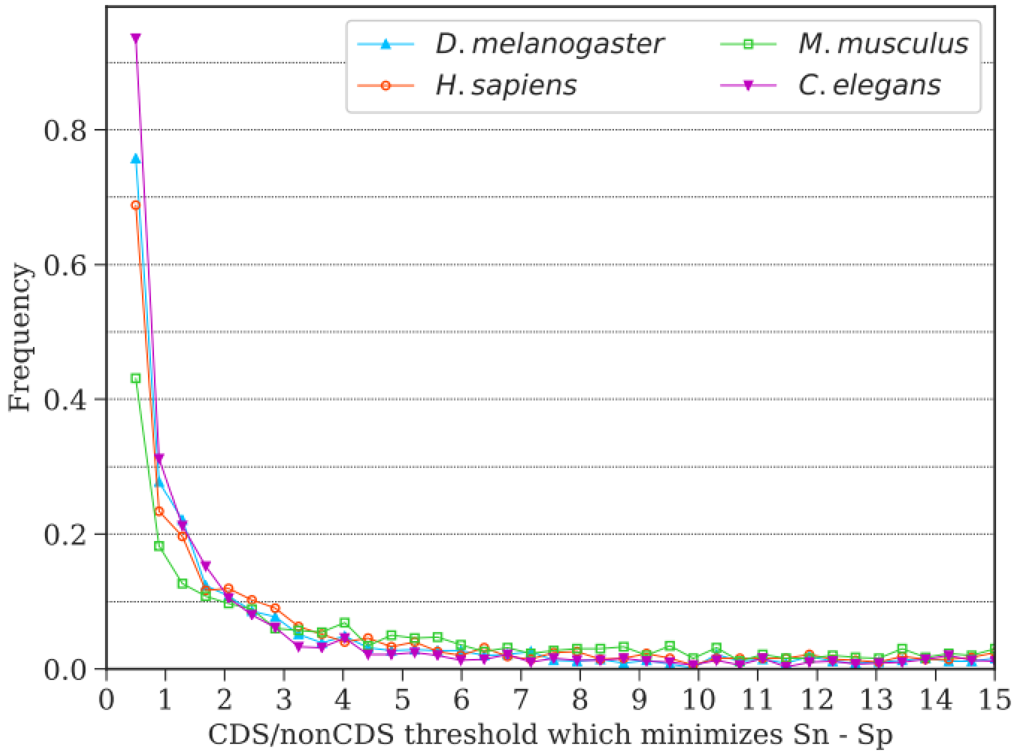

Fig. S9. Distribution of CDS/nonCDS threshold values which attempt to balance Sn and Sp scores on individual genes from the G3PO dataset. Training species is indicated at the top of each pair of plots. Training consisted of 800,000 randomly selected nts with a 50/50 split of coding and non-coding samples.

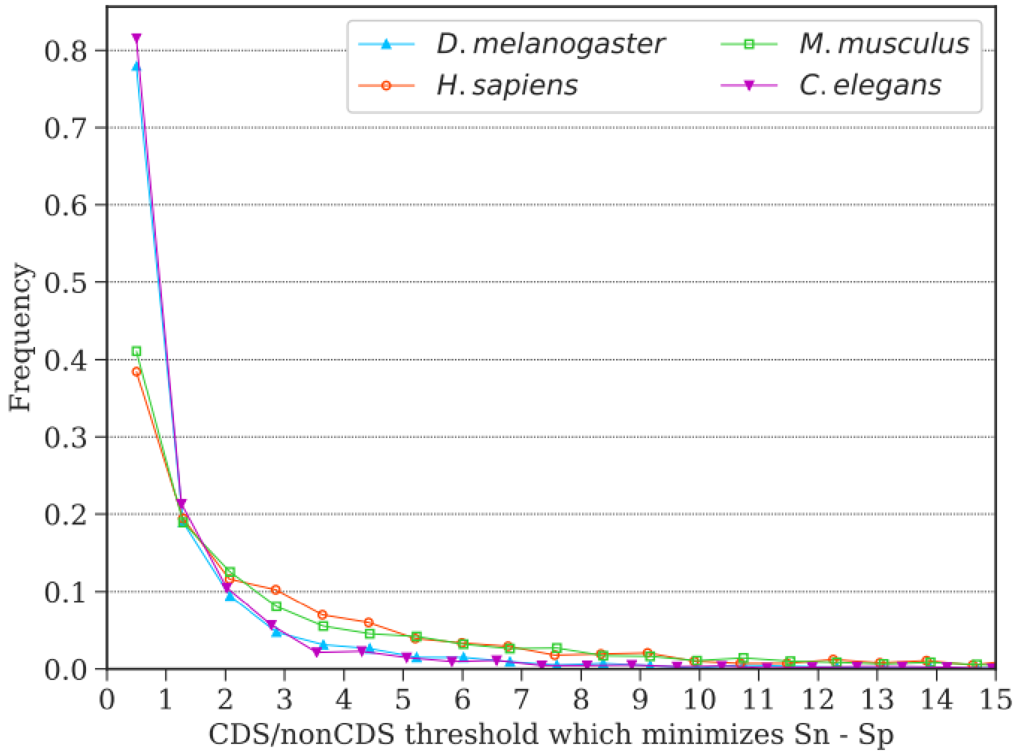

Fig. S10. Distribution of CDS/nonCDS threshold values which attempt to balance Sn and Sp scores on individual genes from the G3PO dataset. Training species is indicated at the top of each pair of plots. Training consisted of 8 million randomly selected nts with a 50/50 split of coding and non-coding samples.
